# Friends with Benefits: An Inside Look of Periodontal Microbes’ Interactions Using Fluorescence In Situ Hybridization—Scoping Review

**DOI:** 10.3390/microorganisms9071504

**Published:** 2021-07-14

**Authors:** Guilherme Melo Esteves, José António Pereira, Nuno Filipe Azevedo, Andreia Sofia Azevedo, Luzia Mendes

**Affiliations:** 1 Faculty of Dental Medicine, University of Porto, Rua Dr. Manuel Pereira da Silva, 4200-393 Porto, Portugal; jpereira@fmd.up.pt (J.A.P.); lgoncalves@fmd.up.pt (L.M.); 2 LEPABE—Laboratory for Process Engineering, Environment, Biotechnology and Energy, Faculty of Engineering, University of Porto, Rua Dr. Roberto Frias, 4200-465 Porto, Portugal; nazevedo@fe.up.pt (N.F.A.); asazevedo@fe.up.pt (A.S.A.); 3 i3S—Instituto de Investigação e Inovação em Saúde, Universidade do Porto, Rua Alfredo Allen 208, 4200-135 Porto, Portugal; 4 IPATIMUP—Institute of Molecular Pathology and Immunology, University of Porto, Rua Júlio Amaral de Carvalho 45, 4200-135 Porto, Portugal

**Keywords:** periodontal diseases, fluorescence in situ hybridization, dental biofilm, imaging, microbes, oral microbiota

## Abstract

Fluorescence in situ hybridization (FISH) has proven to be particularly useful to describe the microbial composition and spatial organization of mixed microbial infections, as it happens in periodontitis. This scoping review aims to identify and map all the documented interactions between microbes in periodontal pockets by the FISH technique. Three electronic sources of evidence were consulted in search of suitable articles up to 7 November 2020: MEDLINE (via PubMed), Scopus (Elsevier: Amsterdam, The Netherlands), and Web of Science (Clarivate Analytics: Philadelphia, PA, USA) online databases. Studies that showed ex vivo and in situ interactions between, at least, two microorganisms were found eligible. Ten papers were included. Layered or radially ordered multiple-taxon structures are the most common form of consortium. Strict or facultative anaerobic microorganisms are mostly found in the interior and the deepest portions of the structures, while aerobic microorganisms are mostly found on the periphery. We present a model of the microbial spatial organization in sub- and supragingival biofilms, as well as how the documented interactions can shape the biofilm formation. Despite the already acquired knowledge, available evidence regarding the structural composition and interactions of microorganisms within dental biofilms is incomplete and large-scale studies are needed.

## 1. Introduction

Periodontitis is a chronic inflammatory disease that results in the loss of the tooth-supporting tissues, which can lead to tooth loss when untreated [1]. It is the consequence of the imbalance between the polymicrobial microbiota, that colonizes the tooth surfaces in form of biofilms, the immune and inflammatory host response within the gingival tissues [1,2,3] and the individual variations in the stock of these taxa [4]. This imbalance, in susceptible individuals, results in the loss of clinical attachment, triggering the formation of periodontal pockets [5,6]. As with other chronic diseases, periodontitis requires supportive care to avoid its recurrence [7]. Furthermore, mounting evidence suggests that many chronic disorders, such as diabetes and cardiovascular diseases, are related to periodontitis via systemic inflammation caused by periodontal bacteria [8].

A total of 2074 genomes and 529 taxa of microbes are estimated to inhabit the oral ecosystem [9]. While most of these microorganisms are not directly associated with periodontitis, they may create the necessary conditions (e.g., nutrient supply or oxygen depletion) for other microorganisms to grow and disrupt the periodontal balance between host and microbes. Microbes’ spatial organization, the understanding of the interactions between microbes in supra- and subgingival biofilms, and how it influences the establishment, development, and the outcome of the periodontal diseases have been emerging subjects in periodontal microbiology.

The development of culture-independent methods has allowed the identification of periodontitis-associated uncultured and fastidious species, providing a more detailed look at the bacterial communities in periodontal tissues. Fluorescence in situ hybridization (FISH) has proven to be particularly useful to describe the microbial composition and spatial organization of mixed microbial infections, in time and space within their natural context. This molecular technique relies on the hybridization of single-stranded, fluorescently labeled DNA-, RNA-, or nucleic acid mimics-targeted oligonucleotides probes with fluorescent molecules (e.g., fluorochromes) that hybridize to its complementary conserved 16S or 23S rRNA sequences in the microorganism [10,11,12].

Even in simple samples where only two or three fluorochromes are used, spectral overlap and crosstalk seem difficult to eradicate when performing standard FISH in multiplex experiments. Additionally, the use of bandpass filters in fluorescence image acquisition restricts the number of fluorochromes that can be simultaneously distinguished, making it problematic to recognize one signal from another simultaneously with certainty and consequently restricting the microscopic identification of various taxa of microbes in single samples [13,14].

To avoid spectral overlap and crosstalk, Valm, A.M. et al. (2012) [14,15] developed a strategy—the Combinatorial Labeling and Spectral Imaging (CLASI)—and blended it with FISH (CLASI-FISH). This technique relies on the labeling of microbes of interest with two or more combinations of fluorochromes [14], increasing spectral discrimination of the fluorochromes that have a propensity to overlap in excitation and emission spectra. CLASI-FISH is currently capable to distinguish unambiguously 120 differently labeled organisms [16], resulting in exclusive mixed colors that are distinguished by the application of linear unmixing algorithms.

This scoping review has the purpose to identify and map the existing evidence about the in situ and ex vivo interactions between microbes in periodontal pockets, identified with the FISH technique, as well as to identify and analyze any knowledge gaps that can point to further research directions.

## 2. Materials and Methods

### 2.1. Focused Question

This scoping review was conducted following the guidelines of the Transparent Reporting of Systematic Reviews and Meta-Analyses extension for scoping reviews (PRISMA-ScR) [17], to answer the focused (PCC—Population, Concept, Context) question: “What are the identified interactions between microbes in periodontal pockets as evaluated by the FISH technique?”

### 2.2. Search Strategy and Information Sources

Three electronic sources of evidence were consulted in search of suitable articles that matched the aim of this review: MEDLINE (via PubMed), Scopus (Elsevier: Amsterdam, The Netherlands), and Web of Science (Clarivate Analytics: Philadelphia, PA, USA) online databases. The databases were consulted up to the 7 November 2020.

Papers were searched using the following keywords: “Periodontal disease”; “Periodont *”; “Periodontal pockets”; “Microbes”; “Oral biofilm”; “Micro *”; “Oral micro *”; “Bacteria”; “Imaging”; “FISH”; “FISH technique”; “fluorescence in situ hybridization” and “hybridization”. The keywords were combined with the Boolean operators “AND” or “OR” with the proximity operators [“” and ()] and with the truncation operator (*) used whenever appropriate. The search strategy was personalized according to the different databases (Table S1).

The electronic database search was supplemented with a hand search across the references of all included papers. The authors of the included papers were contacted to find additional unpublished images that could fulfill our inclusion criteria and were asked permission to reproduce images in this scoping review. When required, additional permission of all reproduced images was granted by the Copyright Clearance Center (Danvers, MA, USA).

### 2.3. Eligibility Criteria

All studies that described ex vivo and in situ interactions between, at least, two microorganisms were found eligible. We only incorporated articles applied in humans.

The exclusion criteria detached experiments using other molecular cytogenetic techniques rather than the FISH technique, articles with no images, or experiments that used manufactured bovine enamel/dentin slabs, acrylic, or epoxy resin appliances to extract dental biofilm. Restrictions were also made to article type excluding reviews, case reports, or letters.

### 2.4. Screening and Selection of Sources of Evidence

Two independent reviewers (G.M.E., L.M.) selected papers by evaluating their titles and their abstracts information. Any disagreements in the acquired results were resolved upon discussion with a third reviewer (A.S.A.). Furthermore, the selected articles were then read in full and were not included if did not fulfill the inclusion criteria or if any of the exclusion criteria was detected. Using the Cohen’s Kappa method and IBM SPSS (Version 26) program, the interrater reliability (IRR) was calculated.

### 2.5. Data Extraction and Analysis

Data from the included articles were processed for analysis. Information regarding the year of publication, study design, FISH conditions, probes’ names and sequences, images, microorganisms found, their location, and their interactions within the biofilm were collected in parallel by G.M.E. and L.M. The data’s interpretation and analysis were debated until a consensus was reached.

### 2.6. Synthesis of Results

The studies were categorized based on the collected data. Evidence is reported in a table and a visual representation, that incorporates all the images in the finest resolution acquired from the FISH experiments performed on the included papers.

## 3. Results

### 3.1. Selection of Sources of Evidence

The initial electronic search resulted in 2090 studies, of which 832 located in PubMed, 625 in Web of Science (Clarivate Analytics: Philadelphia, PA, USA), and 633 in Scopus (Elsevier: Amsterdam, The Netherlands). After removing 1144 duplicated studies, 905 studies were rejected after screening articles by title and abstract. The remaining 41 studies were obtained and analyzed. After full-text reading, 8 studies met the inclusion criteria. Additional hand searching of the reference lists of the selected papers retrieved 10 additional studies for full-text reading, of which 2 papers met the inclusion criteria. As such, 10 papers [3,18,19,20,21,22,23,24,25,26] were included in the present scoping review.

**Figure 1:**
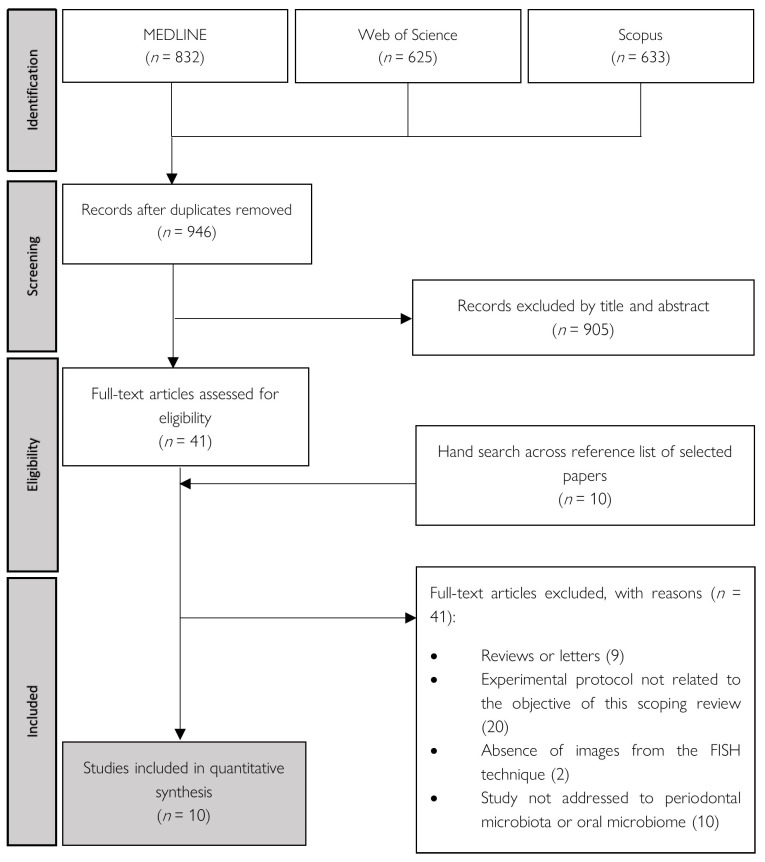
Flowchart of literature search and study selection.

The Cohen’s Kappa method was used to calculate the interrater reliability (IRR) in the selection process by titles and abstracts, which yielded a value of 0.965. The PRISMA flow diagram (Figure 1) demonstrates the selection process.

### 3.2. Characteristics of Sources of Evidence

**Figure 2:**
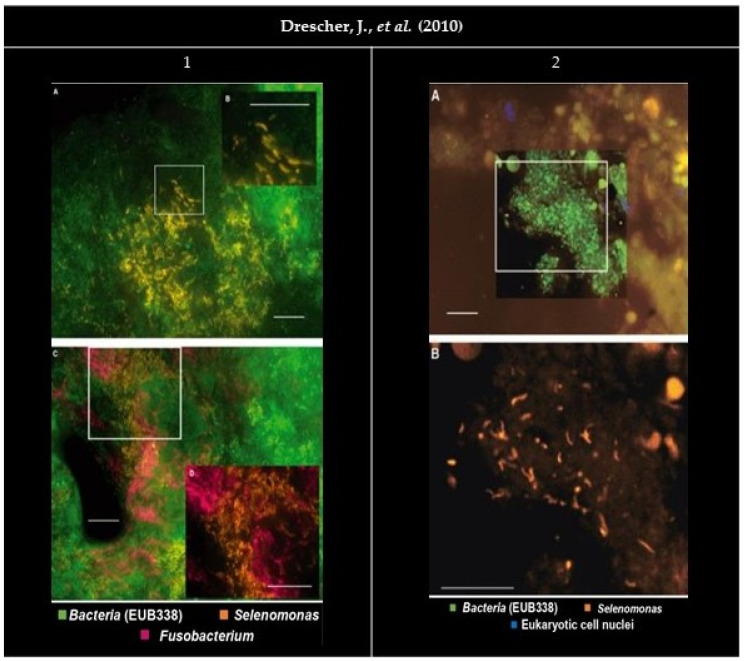
Panel of the gathered images. (**1A**,**C**) Organisms stained by the probe SELE appear as densely packed groups in the cervical portion of the subgingival biofilm. (**1B**) Higher magnification shows a crescent-shaped morphology. (**1C**,**D**) EUB338_FITC_ (Fluorescein isothiocyanate) (green), SELE_Cy3_ (Cyanine 3) (bright orange) and FUSO_Cy5_ (Cyanine 5) (magenta). (**1D**) Higher magnification, with EUB338_FITC_ filter removed for better interpretation. Bars indicate 10 µm. (**2**) FISH (Fluorescence in situ hybridization) performed on a gingival biopsy. (**2A**) EUB338_FITC_ (green), SELE_Cy3_ (orange), and eukaryotic cell nuclei stained with DAPI (4′, 6-diamino-2-phenylindole) (blue). (**2B**) Higher magnification took with the Cy3 filter set only. Bars indicate 10 µm. All images were reprinted and adapted with the publisher’s permission.

**Figure 3:**
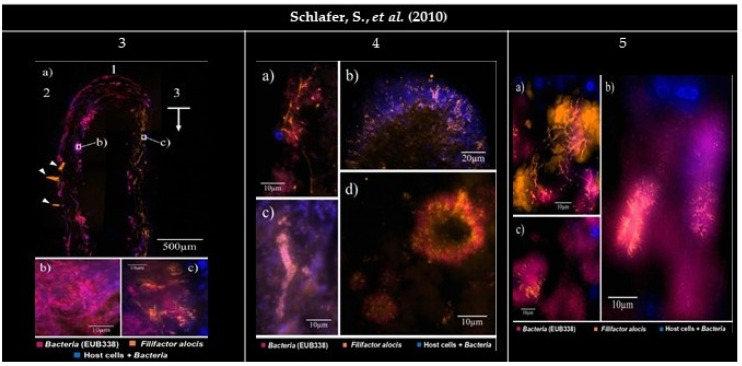
Panel of the gathered images. (**3**) Subgingival biofilm visualized by FISH (Fluorescence in situ hybridization). EUB338_Cy5_ (Cyanine 5) (magenta), FIAL_Cy3_ (Cyanine 3) (bright orange), and DAPI (4′, 6-diamino-2-phenylindole) staining (blue). DAPI stains both host cell nuclei and bacteria. (**3a1**) The carrier tip. (**3a2**) The carrier side facing the tooth. (**3a3**) The carrier side facing the pocket epithelium. (**3a1**,**2**) Little or no presence of *Filifactor alocis*. (**3a3**) Presence of a bright orange signal, indicating a vindicated presence of *F. alocis* (arrow). The image show artifacts caused by the folding of the embedded carriers (arrowheads). (**3b**,**c**) Higher magnification. (**3b**) Rare colonization of *F. alocis* amongst the bacteria. (**3c**) *F. alocis* in densely packed groups among the organisms on the carrier side facing the soft tissues and host cell nuclei (blue). (**4**) Establishment of *Filifactor alocis* in a subgingival biofilm. (**4a**) Overlay of FIAL_Cy3_, EUB338_Cy5_, and DAPI filter sets. In some parts of the biofilm *F. alocis* rods can reach a considerable length. (**4b**,**c**) Overlay of FIAL_Cy3_ and DAPI filter sets. (**4b**) Radial orientation of *F. alocis* towards the exterior of a mushroom-like protuberance. (**4c**) Test-tube-brush formations of *F. alocis* around signal-free channels. (**4d**) Overlay of FIAL_Cy3_ and EUB338_Cy5_ filter sets. *F. alocis* and fusiform bacteria form concentrical structures. (**5**) Establishment of *Filifactor alocis* in periodontal tissue. (**5a**) *F. alocis* forming tree-like structures among coccoid and fusiform bacteria and autofluorescent erythrocytes. (**5b**) *F. alocis* forming palisades with fusiform bacteria around large rod-shaped eubacterial organisms. (**5c**) *F. alocis* being part of concentrical bacterial aggregations such as those found in (**4d**). All images were reprinted and adapted with the publisher’s permission.

**Figure 4:**
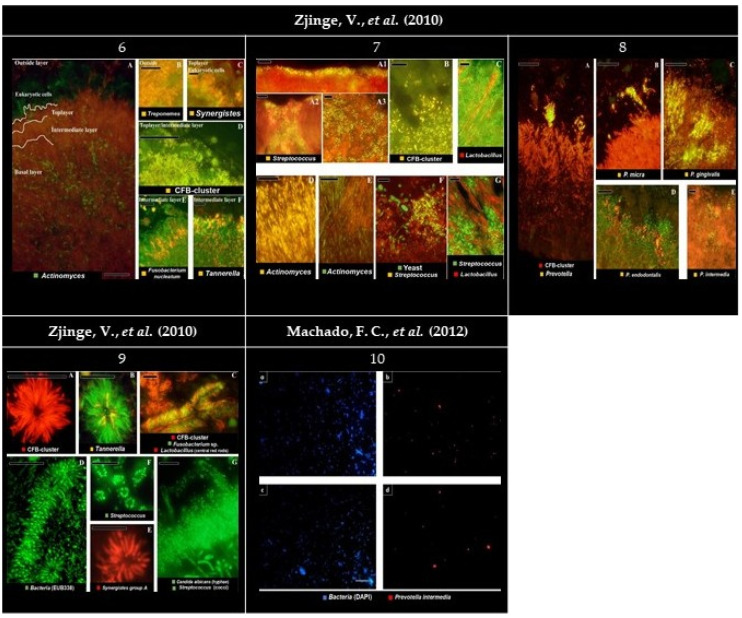
Panel of the gathered images. (**6**) Localization of the most abundant species in subgingival biofilms. (**6A**) Overview of the four layers of subgingival biofilm. *Actinomyces* sp. (green), bacteria (red), and eukaryotic cells (large green cells on top). (**6B**) *Spirochaetes* (yellow) outside the biofilm, without clear organization. (**6C**) Detail of *Synergistetes* (yellow) in the top layer near PMN’s (Polymorphonuclear leukocytes) (green). (**6D**) Presence of the *Cytophaga-Flavobacterium-Bacteroides* cluster (CFB-cluster) (yellow) in the top and intermediate layer. (**6E**) *Fusobacterium nucleatum* in the intermediate layer. (**6F**) *Tannerella* sp. (yellow) in the intermediate layer. Each panel is double-stained with probe EUB338 labeled with FITC (Fluorescein isothiocyanate) or Cy3 (Cyanine 3). Bars indicate 10 µm. (**7**) Localization of the most abundant species in supragingival biofilms. (**7A1**–**C**) The second layer. (**7D**–**G**) Basal layer. (**7A1**–**A3**) *Streptococcus* sp. disposed of in different ways on the second layer. (**7B**) Cells from the CFB-cluster stained in the top layer of the biofilm. (**7C**) *Lactobacillus* sp. (red) through the top layer. (**7D**) On the basal layer, *Actinomyces* sp. cells (yellow). (**7E**) *Actinomyces* sp. (green) and chains of cocci. (**7F**) Colonies of *Streptococcus* sp. (yellow) all-around yeast cells (green) and bacteria unidentified (red). (**7G**) *Streptococcus* sp. (green) growing closely to *Lactobacillus* sp. (red). Black holes might be channels through the biofilm. Panels (**A**–**C**,**E**,**F**) are double stained with probe EUB338 labeled with FITC or Cy3. Bars indicate 10 µm. (**8**) Localization of presumptive species associated with periodontitis in subgingival biofilms. (**8A**) Colonization of the subgingival biofilm by the CFB-cluster species (red) and *Prevotella* sp. (yellow). Since *Prevotella* sp. are part of the CFB-cluster of bacteria, cells appear in yellow. (**8B**) Top of the biofilm with a micro-colony of *Parvimonas micra* (yellow). (**8C**,**D**) Micro-colonies of *Porphyromonas gingivalis* (yellow) and *Porphyromonas endodontalis* (yellow) in the top layer, respectively. (**8E**) Micro-colonies of *Prevotella intermedia* in the top layer. Panels B, C, D, and E are double stained with probe EUB338 labeled with FITC or Cy3. Bars indicate 10 µm. (**9**) Bacterial aggregates detected in both sub- and supragingival plaque. (**9A**) Filamentous cells from the CFB-cluster in the fourth layer of the subgingival plaque. (**9B**) *Tannerella* sp. (yellow) in a test-tube brush. (**9C**) Test-tube brush with *Lactobacillus* sp. (red rods) as central structures. *Fusobacterium nucleatum* (green) and CFB-cluster filaments (red), morphologically identical to *Tannerella* forsythia, perpendicularly radiating around lactobacilli. (**9D**) Test tube brush stained with the eubacterial probe. (**9E**) *Synergistetes* group A species forming aggregates solely with themselves. (**9F**) *Streptococcus* sp. (green) aggregation around a central cell (not stained) in supragingival plaque. (**9G**) Supragingival plaque with *Streptococcus* sp. (green cocci) adhering to a central axis of yeast cells or hyphae, such as *Candida albicans* (green hyphae). Bars indicate 10 µm. (**10a**,**c**) Plaque specimen stained for total bacterial cells with DAPI (4′, 6-diamino-2-phenylindole) in pregnant and non-pregnant women, respectively. (**10b**,**d**) Plaque specimen stained for *Prevotella intermedia* (with probe Pint649) in pregnant and non-pregnant women, respectively. Bars indicate 20 µm. All images were reprinted and adapted with the publisher’s permission.

The general characteristics of the six case series, the three case-control studies, and the one cross-sectional study are presented in this section (Table 1). We divided certain images into three panels to make the analysis of the gathered images simpler (Figure 2, Figure 3 and Figure 4). Due to the well-defined approach, we paid special attention to the results of one particular study (Mark Welch, J.L., et al.).

In Mark Welch, J.L., et al. (2016) [18] only 13 out of 57 genera tested had at least 3% mean abundance and were also prevalent, being identified in more than 90% of supragingival specimens (*Corynebacterium* sp., *Capnocytophaga* sp., *Fusobacterium* sp., *Leptotrichia* sp., *Actinomyces* sp., *Streptococcus* sp., *Neisseria* sp., *Haemophilus*/*Aggregatibacter* sp., *Porphyromonas* sp., *Rothia* sp., *Lautropia* sp., *Veilonella* sp., and *Prevotella* sp.).

*Corynebacterium* sp. was remarkably specific to supragingival (12%) and subgingival (8%) plaque. By contrast, genera such as *Streptococcus* sp., *Veillonella* sp., and *Haemophilus* sp. occupied a wide range of substrates in oral ecosystems.

**Figure 5:**
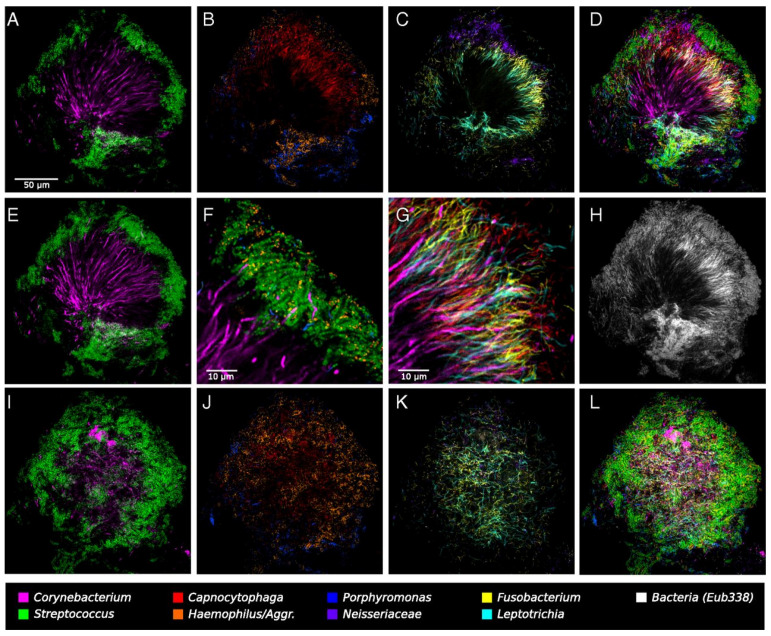
Hedgehog-shape structure. (**A**–**D**,**F**–**H**) represents a single focal plane nearby the middle part of the structure. (**A**) *Corynebacterium* sp. filaments radiating outward from near the center of the image and coccoid *Streptococcus* sp. cells arranged around the distal tips of the *Corynebacterium* sp. filaments. (**B**) Cells of *Haemophilus/Aggregatibacter* sp. and *Porphyromonas* sp. sited at the structure’s periphery, in the same region as the *Streptococcus* sp. cells. *Capnocytophaga* sp. also inhabited a wideband inside the periphery. (**C**) *Fusobacterium* sp. and *Leptotrichia* sp. occupying this band called “filament-rich halo”. *Neisseriaceae* establishing constellations in and nearby the periphery. *Actinomyces* sp. represented by a small number of cells located near the structure’s base. (**D**) All taxa overlayed. Many cells from the “filament-rich annulus” overlapped the exterior of the consortia. (**E**–**G**) Detail of the *Corynebacterium*’s arrangement relatively to other taxa. (**E**) The maximum intensity projection of three adjacent optical sections shows that these filaments are continuous from the center to the periphery of the structure for more than 50 µm. Some filaments persist visible within the *Streptococcus* sp. cells. (**F**) Detail of the periphery. Corncob structures are composed of a filamentous core (sometimes visualized as *Corynebacterium* filaments but often not stained) bordered primarily by *Streptococcus* sp. cells but also by *Porphyromonas* sp. and *Haemophilus/Aggregatibacter* sp., both in proximal contact with *Streptococcus* sp. cells. (**G**) On the periphery of these corncob structures, *Corynebacterium* sp. filaments pass through the halo that is highly densely colonized with elongated rods of *Fusobacterium* sp., *Leptotrichia* sp., and *Capnocytophaga* species. (**H**) The fluorescent signal of the universal probe EUB338. (**I**–**L**) The exterior of the hedgehog structure, composed mainly of corncobs. (**K**) The edge of the *Fusobacterium*-*Leptotrichia* sp. halo. Reprinted with the publisher’s permission.

A complex microbial consortium in a hedgehog-shaped structure was observed, showing the spatial organization of the plaque microbiota. In short, these hedgehog structures were radially organized, with a multi-taxa consortium composed of a skeleton mainly of *Corynebacterium* sp. with *Streptococcus* sp. cells arranged around the distal tips, a multi-genus filament-rich halo composed of *Fusobacterium* sp., *Leptotrichia* sp., and *Capnocytophaga* sp. cells, and a periphery of corncobs structures composed by a filamentous core bordered primarily with *Streptococcus* sp. cells, *Porphyromonas* sp. and *Haemophilus*/*Aggregatibacter* sp. (Figure 5).

**Figure 6:**
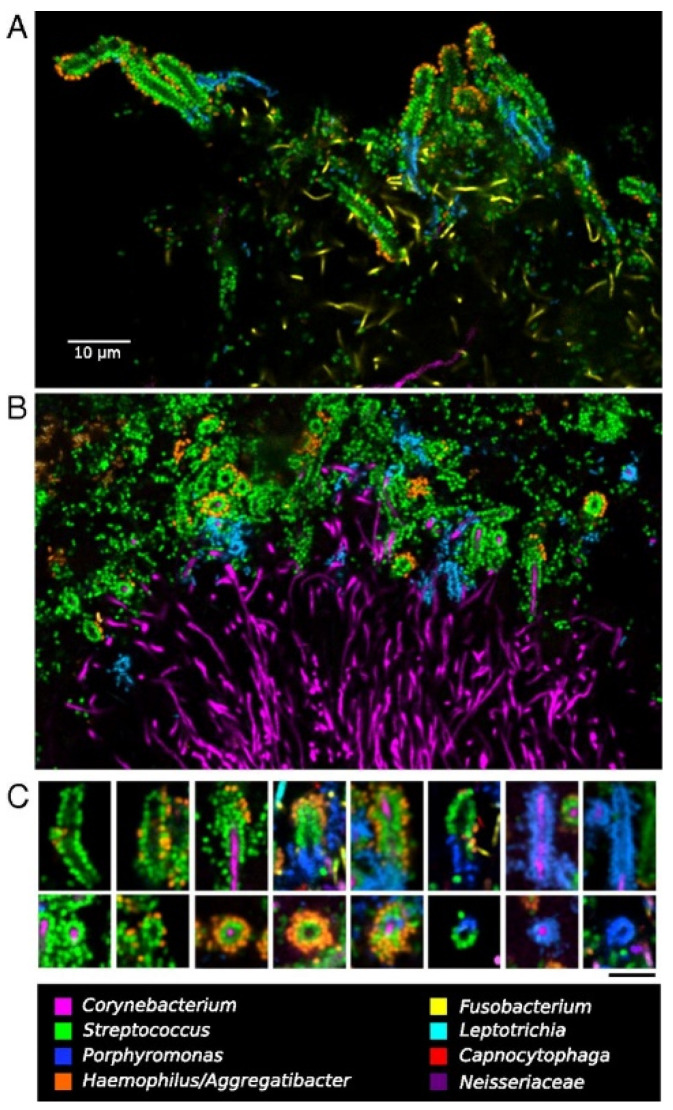
Dense corncob structures in supragingival plaque. (**A**) The central filament lacked hybridization in whole-mount preparation. (**B**) The central filament was well visualized in methacrylate-embedded and sectioned preparations. (**C**) Representative images of types of corncobs found. Bar indicates 5 µm in (**C**). Reprinted with the publisher’s permission.

The corncobs at the periphery showed that “kernels” (coccoid cells) were composed of different taxonomic types and could be either single or double layered (Figure 6). Single-layer corncobs had coccoid cells of both *Streptococcus* sp. or *Porphyromonas* sp. (in some cases *Porphyromonas* sp. kernels coexisted with *Streptococcus* sp. around the same filament), whereas double-layer kernels consisted of a combination of *Streptococcus* sp. in the inner layer and *Haemophilus*/*Aggregatibacter* sp. in the outer layer.

The most common type of kernels visualized were the ones that had a single layer of *Streptococcus* sp. cells surrounded by a partial or complete layer of *Haemophilus*/*Aggregatibacter* species. *Porphyromonas* sp. cells were only observed organized in single-layer corncobs. On contrary, cells of the genus *Haemophilus*/*Aggregatibacter* sp. were only found forming double-layer structures, exclusively with *Streptococcus* sp. cells, demonstrating an undoubtedly specific relationship. Competitive, exploitative, or mutualistic interactions between these taxa were detected in this specific biofilm architecture between single- and double-layers corncobs.

Within hedgehogs, bacteria do not form broad single-taxon groups; rather, cells were observed intermingling with at least four different taxa. The authors only did not find this type of interaction between *Actinomyces* sp. and *Corynebacterium* sp. cells, where were visualized irregular clumps in the base of the hedgehogs or nearby the hedgehogs, rather than intermixed within this structure.

Hedgehogs were the most observed type of structure. Some samples had multiple hedgehogs’ structures adjacent to each other (Figure S1), especially on the tooth surface on the buccal side and plaque from the gingival margin. Another kind of consortia was also found: a cauliflower structure in plaque (Figure S2) constituted by *Lautropia* sp., forming the center of the structure, surrounded by *Streptococcus* sp., *Haemophilus*/*Aggregatibacter* sp., and *Veilonella* species. Dispersed cells of *Prevotella* sp., *Rothia* sp., and *Capnocytophaga* sp. were also visible.

The findings collected from the included articles in this scoping review are summarized in Table 2. We also included a supplemental table, with details on all the oligonucleotide probes and the FISH conditions used in each microorganisms’ analysis (Table S2).

## 4. Discussion

### 4.1. Summary of Evidence

Our findings from the obtained images indicate a paucity of research focusing specifically on the study of the spatial organization of periodontal pathogens within oral biofilms from the *Bacteria* domain and its etiologic significance.

In our results, the biofilm’s fluorescent intensity and variety of labeled microorganisms increased as images drifted from the tooth to the epithelium side, and from the pocket’s depth to the coronal surface, indicating differences in the physiological activity of the cells. Subsequently, we present three possible explanations: (i) the cell structures and morphologies of supragingival biofilms are much more diverse than those of subgingival biofilms; (ii) the unidentified microorganisms may belong to species for which there are no probes available (iii) prior stages of the biofilm that have been shielded from nutrients, comprising dead/inactive cells with low fluorescence activity, are located in the basal layers [26].

Although the stock of the oral microbiota has a lot of inter-and intra-individual variation, about 16 genera are almost universally found in supragingival dental biofilm, including *Corynebacterium* sp., *Capnocytophaga* sp., *Fusobacterium* sp., *Leptotrichia* sp., *Actinomyces* sp., *Campylobacter* sp., *Streptococcus* sp., *Neisseria* sp., *Selenomonas* sp., *Haemophilus*/*Aggregatibacter* sp., *Porphyromonas* sp., *Rothia* sp., *Lautropia* sp., *Veilonella* sp., and *Prevotella* sp. [18,23,26,27,28]. Bacteria that thrive below the gumline are cut off from high oxygen stress, salivary and dietary nutrients, relying on gingival crevicular fluid (GCF) for nutrition. As a result, strict or facultative anaerobic proteolytic bacteria become more prevalent in the subgingival microbiota, such as *Filifactor* sp., *Fusobacterium* sp., *Parvimonas* sp., *Porphyromonas* sp., *Prevotella* sp., *Tannerella* sp., and *Treponema* sp. [3,24,25,26,29,30], particularly in individuals with periodontitis.

In healthy people, the fungal load is thought to be lower than the bacterial load. However, the size and morphology of fungal cells, as well as their synergistic interactions with bacteria, indicate that these species play an important role in the formation of dental biofilms [26,31,32].

The adsorbing of salivary proteins and glycoproteins to the tooth’s surface triggers the initial plaque establishment, forming the acquired enamel pellicle (AEP)—a conditioning layer that covers all teeth present in the oral cavity and acts as a substrate for bacterial attachment [33,34]. The AEP is formed during the early stages of teeth eruption when saliva contacts the tooth’s surface [34] and is never fully removed even if professional dental hygiene is performed.

Planktonic cells, aggregates of cells, and early colonizers (e.g., *Streptococcus* sp., *Lactobacillus* sp., *Actinomyces* sp., and *Candida* sp.) adhere to this pellicle via specialized adhesins on the bacterial cell surface [35,36]. These species do not promiscuously bind to any filament available, but rather engage in a highly specific interaction with already adhered cells, such as *Corynebacterium* sp., *Actinomyces* sp., [18], or yeast/hyphae cells forming the first layer of supragingival biofilm [26,31].

The role of *Actinomyces* sp. as a primary colonizer has already been proved due to its significant role in gingivitis [37]. Many *Actinomyces* species, such as *A. naeslundii*, *A. oris*, and *A. johnsonii* [38] have been found in supra- and subgingival biofilms’ specimens from diseased patients, suggesting that these are the most significant biofilm initial formers among the *Actinomyces* genus. *Actinomyces* sp. can store intracellular glycogen or search for biofilm material such as extracellular polymeric substances and compounds from dead bacterial cells [26,39], which can create an important advantage in surviving in the deepest layers of the biofilm community. *Actinomyces* sp. were found in the base of the hedgehogs’ structures [18], which also suggests that *Corynebacterium* sp. cells do not directly colonize the tooth surface but on a previous and established biofilm containing *Actinomyces* species.

Possibly, these already adhered microorganisms may create a microenvironment favorable to other colonizers to grow. Therefore, biofilm maturation occurs by the coaggregation of planktonic bacteria to an already adhered biofilm [40].

Early colonizers of supragingival dental surfaces are usually facultative anaerobic bacteria, such as *Streptococcus* sp. These species produce carbon dioxide (CO_2_), lactate, and acetate, containing hydrogen peroxide (H_2_O_2_) by consuming oxygen (O_2_). The redox potential is lowered, allowing strict anaerobes (e.g., *Fusobacterium* sp., *Leptotrichia* sp., and *Capnocytophaga* sp.) to settle and multiply in the biofilm.

**Figure 7:**
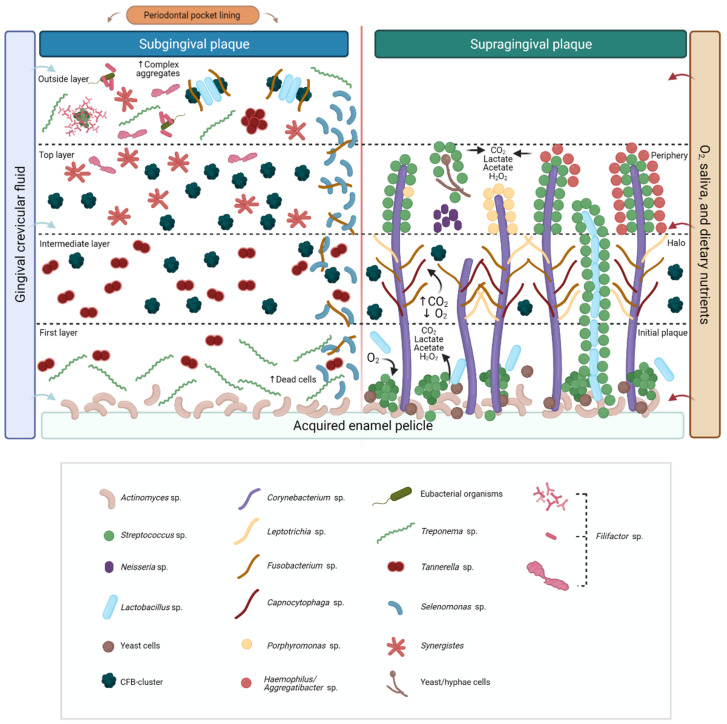
Summary hypothesis for the interpretation of the gathered evidence. Microorganisms that thrive in the subgingival environment, in contrast to bacteria in supragingival biofilm, are cut off from high oxygen stress, salivary, and dietary nutrients, depending on gingival crevicular fluid (GCF) for nutrition. The adsorbing of salivary proteins and glycoproteins to the tooth’s surface triggers the initial plaque establishment, forming the acquired enamel pellicle (AEP)—a conditioning layer for bacterial attachment. In the supragingival biofilm, *Corynebacterium* sp. filaments bind to an existing biofilm containing *Streptococcus* sp., *Actinomyces* sp., yeast cells, and *Lactobacillus* sp. These species produce carbon dioxide (CO_2_), lactate, and acetate, containing hydrogen peroxide (H_2_O_2_) by consuming oxygen (O_2_). The redox potential is lowered, allowing strict anaerobes (such as *Fusobacterium* sp., *Leptotrichia* sp., and *Capnocytophaga* sp.) to settle and multiply in the CO_2_-requiring “halo” section. This environment is also favorable to the growth and development of microorganisms from the *Cytophaga-Flavobacterium-Bacteroides* cluster (CFB-cluster), especially, *Prevotella* sp. In the periphery, cells shape single- or double-layered corncobs structures. *Streptococcus* sp. adheres to a central axis of yeast/hyphae, *Corynebacterium* sp., or *Lactobacillus* sp. cells. Subgingival biofilm is divided into four layers: (i) the first layer, which is made of early colonizers displaying few fluorescences; (ii) the intermediate and (iii) the top layers are composed of a variety of microorganisms from the CFB-cluster, *Treponema* sp., *Tannerella* sp., *Synergistetes*, and *Fusobacterium* sp. tangled with *Selenomonas* sp. in the cervical portion; (iv) the outside layer, made of Treponemes nearby a complex mixture of CFB-cluster, *Lactobacillus* sp. and *Fusobacterium* sp. cells coaggregated in fine test-tube brushes. *Tannerella* sp. and *Synergistetes* formed aggregates solely with themselves. In this last layer, *Filifactor* sp. form a variety of concentrical-orientated structures, tree-like shapes among coccoid cells, palisades around eubacterial organisms, or remote test-tube-brush shapes.

The presence of *Streptococcus* sp. and bacteria from the *Cytophaga-Flavobacterium-Bacteroides* cluster (CFB-cluster) in the second layer of supragingival biofilm [26] could indicate a critical transition of a supragingival biofilm made of predominantly Gram-positive saccharolytic bacteria to a Gram-negative proteolytic subgingival biofilm, which could be caused by nutrient availability (e.g., dietary sugars in the supragingival biofilm, or proteins from saliva and GCF in the subgingival biofilm). Additionally, the presence of *Streptococcus* sp. in the basal and second layers of biofilm also allows us to infer that these organisms can adapt to a wide variety of environments, settling first as early colonizers but with the ability to expand and prosper. Our hypothesis is summarized in Figure 7.

The presence and abundance of *Corynebacterium* sp. in hedgehog structures in a bush-like skeleton, anchored from the presumed tooth surface [18,41] may also indicate that this genus plays a key role in the biofilm community, while its biofilm specificity indicates that it occupies a niche that is influenced by tooth surface and/or GCF properties.

By reducing the flow of GCF with the use of anti-inflammatory agents the outgrowth of proteolytic bacteria would be prevented. Another alternative would be to use oxygenating or redox agents to make the gingival crevice less anaerobic, selectively inhibiting the growth of obligate anaerobes [42]. Therefore, the conventional periodontal treatment approaches that involve mechanical removal of biofilm, physical disruption of biofilm structure, or antibiotic therapies could be enhanced.

### 4.2. Microbes’ Herd Mentality Behavior

Taxa that are present primarily or exclusively in one site may provide clues to the distinctive features of the habitat and the role that those taxa contribute to the site but it’s still unclear if pathogens found at the control sites are part of the local/commensal flora or are originated from adjacent periodontal lesion sulcus crevicular fluid [24].

Depending on which partner the microorganism was found coaggregated with, *F. alocis* shaped several conformations, especially with fusiform bacteria, which formed concentrical radial-orientated structures in mushroom-shaped biofilms or palisades structures when the coaggregation also occurred with eubacterial organisms [3]. When isolated, *F. alocis* formed test-tube brushes shapes around signal-free channels. This pattern and degree of organization can play a role in the events that occur during biofilm growth and maturation and be strongly linked to biofilm formation.

Our findings propose that other microorganisms adopted different spatial arrangments influenced by their peers in different biofilm areas and tissues. Test-tube brushes shapes were once again found in a complex mixture of cells containing *T. forsythia*, *Campylobacter* sp., *P. micra*, *Fusobacterium* sp., and *Synergistetes* group A in the outside layer of subgingival biofilm [26]. On the other hand, *Synergistetes* group A adopted a wide cigar-like shape in a palisade lining when found isolated, forming aggregates exclusively with themselves. The CFB-cluster also manifested different shapes, especially *Prevotella* sp. that formed micro-colonies in the top layer of subgingival biofilm but were observed as filamentous, rod-shaped, and coccoid on the intermediate layer.

Depending on which microorganisms they are coaggregated with, some microorganisms appear to “metamorphose”. We call this process microbes’ “herd mentality behavior” and we believe that might be an important advantage of periodontal pathogens. To verify our hypothesis, future research should focus on the spatial organization of periodontal microbiota. The studies should be performed considering sample collection and preparation that preserves as much spatial organization as possible, such as whole-mount preparations that permit the imaging of 3D structures [18].

### 4.3. Limitations

None of the included articles classified periodontal diseases according to current diagnostic criteria. In June 2018, the American Academy of Periodontology (AAP) released a series of reviews in partnership with the European Federation of Periodontology (EFP) [7,43,44,45,46] suggesting significant updates in periodontal diagnose, such as combining chronic and aggressive periodontitis in unique-entity periodontitis with various phenotypes. Another major improvement was the association of the periodontal disease’s classification with its progression rate and prognostic factors in a more accurate and reliable staging classification [47].

The results demonstrate that the available evidence relies on a limited number of observations. Assuming that these observable interactions are somehow representative of the periodontal microbiota in health and disease, is a leap of faith that needs confirmation in large-scale studies. Some images catch our eye, but in the absence of meticulous cell counting, it may contribute to an overestimation of microbes’ population in biofilm specimens, contradicting quantitative findings. This raises the question of whether all FISH studies should involve quantitative cell counting or dynamic time lap observations.

We did not include the identification and screening of gray literature in the scoping review process. However, all authors of the included publications in this review were requested to submit unpublished images and/or articles that matched the review’s goal.

## 5. Conclusions and Future Perspectives

Supragingival biofilm seems to present a radially ordered multiple-taxon structure, backboned by facultative anaerobic long rods, such as *Corynebacterium* sp. and *Lactobacillus* sp., bordered by coccoid and filamentous cells. On the other hand, subgingival biofilm seems to present a more layered multiple-taxon structure from the tooth’s surface to the pocket’s epithelium. Anaerobic microorganisms dominate the subgingival environment, especially in the deepest portions. Outside layers with increased microbial diversity were found in both sub- and supragingival biofilms when compared with the inner layers, and the largest contrast was observed in the subgingival biofilm. Consumers and producers of certain metabolites tend to be spatially related; some microorganisms exhibited the ability to shape various structures influenced by their peers in different biofilm areas. We referred to this last phenomenon as “microbes’ herd mentality behavior” and we believe that it may represent an imperative benefit of certain members of the periodontal microbiota.

In vitro studies or studies performed in animals provided the foundations for the molecular pathology reasoning discussed in this scoping review. We suggest that future research should focus on studying species networks in a more realistic and holistic setting, using complex community-like structures as a model.

In conclusion, and in response to the previous query, the FISH technique was able to detect interactions between microorganisms from the three domains of life, enabling the construction of a possible hypothesis for understanding the influence of these interactions in the establishment and development of periodontal diseases. However, the evidence about the structural composition and microorganisms’ interactions in supra- and subgingival biofilms is scarce and no definitive conclusion can be drawn. The enhanced FISH-based techniques provide significant information about the spatial organization of a complex natural polymicrobial community, allowing researchers and dental medicine doctors to better understand how the individual taxa interact within a community in periodontal diseases, and how their relations compromise the whole assemblage.

## Figures and Tables

**Table 1:** Characteristics of the included studies, such as location of the sampled dental biofilm, exclusion criteria, characteristics of the controls and/or cases, and the purpose of each study. The abbreviations can be found at the bottom of the table.

**Case Series (6)**							
**Authors (Year)**	**Sample**	**Exclusion Criteria**	**Cases**				**Aim**
			**Characteristics**		***n***		
Wecke, J., et al. (2000)	Subgingival	Chronic diseases; antimicrobial therapy within the past 6 months	PPD means of 8.07 ± 1.63 mm		52		Assess the frequency and spatial organization of specific treponemes in RPP
Gmür, R., et al. (2004)	Supragingival	Chronic diseases; Antimicrobial therapy within the past 3 months	Gingival pain, gingival ulcers or necrosis, pseudomembranes, fetid odor, loss of gingival papillae, PPD ≤ 4 mm, having at least 20 natural teeth, BOP, and plaque index		42		Compare the distribution of periodontal pathogens in gingivitis and NUG
Gmür, R. and Lüthi-Schaller, H. (2007)	Subgingival	NAI	Patients with ACP		NAI		Develop an IF-FISH protocol
Schlafer, S., et al. (2010)	Subgingival	Chronic diseases; Antimicrobial/anti-inflammatory therapy within the past 6 months; pregnant or lactating women	GAP: having a disease onset estimated at <30 years and PPD ≥ 6 mm at more than 3 permanent teeth (other than first molars or incisors)		11		Study the architectural function of *Filifactor alocis* in GAP
Zjinge, V., et al. (2010)	Supra- and subgingival	Chronic diseases; antimicrobial therapy within the past 3 months	PPD > 6 mm RBL > 30%		10		Study the biofilm architecture of predominant periodontal taxa
Mark Welch, J.L., et al. (2016)	Supragingival	Subjects suffering from chronic diseases	Subjects refrained from oral hygiene for 12 to 48 h before sample collection		22		
**Case-Control Studies (3)**							
**Authors (Year)**	**Sample**	**Exclusion Criteria**	**Cases**		**Controls**		**Aim**
			**Characteristics**	***n***	**Characteristics**	***n***	
Moter, A., et al. (1998)	Subgingival	Chronic diseases; antimicrobial/anti-inflammatory therapy within the past 6 months	PPD ≥ 6 mm BOP	200	Site clinically not affected	44	Assess the frequency of specific treponemes in RPP
Lepp, P.W., et al. (2004)	Subgingival	Chronic diseases; antimicrobial therapy within the past 3 months; pregnant or lactating women; diabetes or HIV-positive	Gingivitis (BOP; CAL ≤ 1 mm; PPD ≤ 4 mm); Slight periodontitis (BOP; CAL 2–3 mm; PPD ≥ 4 mm); Moderate periodontitis (BOP; CAL 4–5 mm; PPD ≥ 4 mm); Severe periodontitis (BOP; CAL ≥ 6 mm; PPD ≥ 4 mm)	167	Healthy (BOP; CAL ≤ 1 mm; PPD ≤ 3 mm)	67	Identify populations of *Archaea* in periodontal pockets
Drescher, J., et al. (2010)	Subgingival	Chronic diseases; antimicrobial/anti-inflammatory therapy within the past 6 months; pregnant or lactating women	GAP: having a disease onset estimated at <30 years and PPD ≥ 6 mm at more than 3 permanent teeth (other than first molars or incisors); CP: PPD of ≥4 mm at 30% or more of the residual teeth	144	PR subjects: (age ≥ 65 years; at least 20 natural teeth; CAL ≤ 2 mm; PPD ≤ 5 mm)	19	Study the spatial organization of *Selenomonas* sp. in GAP
**Cross-Sectional Study (1)**							
**Authors (Year)**	**Sample**	**Exclusion Criteria**	**Subjects** **’ Condition**		***n***		**Aim**
Machado, F.C., et al. (2012)	Subgingival	Chronic diseases; antimicrobial/psychotropic/anticonvulsant therapy within the past 3 months; professional tooth-cleaning in the previous 6 months or were receiving orthodontic treatment	Pregnant		20		Study the qualitative/quantitative differences of eight periodontal pathogens
			Non-pregnant		20		

ACP: advanced chronic periodontitis; BOP: bleeding on probing; CAL: clinical attachment loss; CP: chronic periodontitis; GAP: generalized aggressive periodontitis; IF-FISH: immunofluorescence-fluorescence in situ hybridization; NAI: no available information; NUG: necrotizing ulcerative gingivitis; PPD: probing pocket depth; PR: periodontitis-resistant; RBL: radiographic bone loss; RPP: rapidly progressive periodontitis.

**Table 2:** A summary in terms of the location where the dental biofilm was collected, the microorganisms detected, their shape and spatial organization within the biofilm, the most relevant interactions detected, and the microscopic technique used in each study. The abbreviations can be found at the bottom of the table.

Authors (Year)	Site	Microorganisms	Shape and Spatial Organization of Microorganisms	Relevant Interactions	Microscopic Technique
Moter, A., et al. (1998)	SUBG	Group I of oral treponemes	Large and dense	These organisms are present in high proportions in subgingival plaque samples and thus represent the predominant flora. Group I treponemes outnumbered group II treponemes. All the treponemes identified predominated at diseased sites but were found infrequently at periodontally stable sites	Dark-field
		Group II of oral treponemes	Thin, slender, wavily		
Wecke, J., et al. (2000)	SUBG	Group I of oral treponemes	Large and undulated	Treponemes appeared spread between Gram-negative bacteria in the deepest parts of the periodontal pockets. Gram-positive cocci were located on the most coronal section of the specimens	CLSM
		*Bacteria*	Rods and coccoid		
Lepp, P.W., et al. (2004)	SUBG	*Methanobrevibacter oralis*	Diplococcobacilli	Treponemal rDNA was found in significantly lower abundance in sites with archaeal rDNA than in sites without archaeal rDNA	CLSM
Gmür, R., et al. (2004)	SUPG	*Leptotrichia buccalis*	Wide and segmented fusiform rods	There was a significantly increased total abundance of periodontal pathogens in the NUG group compared with the gingivitis group	Dark-field
		Indistinguishable *Fusobacterium nucleatum, Capnocytophaga* sp., and *Fusobacterium periodonticum*	Smaller, thin, spindle-shaped, and dotted fusiform rods		
Gmür, R. and Lüthi-Schaller, H. (2007)	SUBG	*Tannerella forsythia*	Clumps	*Tannerella forsythia* was detected in the deepest zones of the periodontal pockets	Epifluorescence
Drescher, J., et al. (2010)	SUBG	*Selenomonas* sp.	Densely packed groups with a crescent-shaped structure from the cervical section to the biofilm portion derived from the pocket’s depth and both on the side facing the tooth and the side facing the soft tissue	These genera appeared spatially related and tangled with each other	Epifluorescence
		*Fusobacterium* sp.	Densely groups with a fusiform shape		
Schlafer, S., et al. (2010)	SUBG	*Filifactor alocis*	(i) Short rod clustered in radial-orientated structures nearby fusiform bacteria on mushroom-shaped biofilms; (ii) Test-tube brush shapes; (iii) Branch-like structures in gingival tissue; (iv) Palisades structures nearby fusiform bacteria and eubacterial organisms	*F. alocis* was present in areas that corresponded to the depth of the pockets, but very occasionally in areas that corresponded to the cervical portion and the carrier’s very tip. *F. alocis* colonized the carrier side facing the soft tissue in most cases and was present in small numbers or not at all on the carrier side facing the root	Epifluorescence
Zjinge, V., et al. (2010)	SUBG-FL	*Actinomyces* sp	Rod-shaped	Display little fluorescence	Epifluorescence
	SUBG-IL	*Fusobacterium nucleatum*	Fusiform cells	TL and a portion of the IL were mostly made of filamentous, rod-shaped, or even coccoid bacteria from the CFB-cluster	
		*Tannerella forsythia*			
		*Tannerella* sp.			
		CFB-cluster	Filamentous, rod-shaped, coccoid		
	SUBG-TL	CFB-cluster	Filamentous, rod-shaped, coccoid, micro-colonies (*Prevotella*)		
		*Synergistetes* group A	Wide cigar-like bacteria in a palisade lining		
		*Parvimonas micra*	Micro-colonies		
	SUBG-OL	Treponemes	Test-tube brush shapes	CFB-cluster cells were found perpendicularly arranged around *Lactobacillus* sp. in test-tube brush shape. Test-tube brushes shapes were also found in a complex mixture of cells	
		CFB-cluster			
		*Lactobacillus* sp.	Rod-shaped		
		*Porphyromonas gingivalis*	Micro-colonies		
		*Porphyromonas endodontalis*			
		*Tannerella forsythia*	Test-tube brush shapes		
		*Campylobacter* sp.			
		*Parvimonas micra*			
		*Fusobacterium* sp.			
		*Synergistetes* group A			
	SUPG-BL	*Actinomyces* sp.	Rod-shaped	Bacterial deposits made up of early colonizers, growing perpendicularly to the tooth surface	
		*Actinomyces* sp. + chains of cocci	Rod-shaped and coccoid		
		*Streptococcus* sp. + yeast and not identified bacteria	Filamentous		
		*Streptococcus* sp. + *Lactobacillus* sp.			
	SUPG-SL	*Streptococcus* sp.	(i) Thin coat; (ii) Colonizing biofilm’s cracks; (iii) Without clear organization	Corncob structures consisting of *Streptococcus* sp. adhering to a central axis of yeast/hyphae cells	
		CFB-cluster	Heterogenous and without clear organization		
		*Lactobacillus* sp.	Long string-shape		
Machado, F.C., et al. (2012)	SUBG	*Prevotella intermedia*	Patchy groups	*P. intermedia* was frequently found in the plaque of pregnant women	Epifluorescence
Mark Welch, J.L., et al. (2016)	SUPG	*Corynebacterium* sp.	Continuous filaments from the base to the periphery of the structure	*Corynebacterium* sp. filaments were crusted at their distal tips by brilliant cocci	CLSM
		*Streptococcus* sp.	Coccoid		
		*Capnocytophaga* sp.	Filamentous	Part of a multi-genus halo	
		*Fusobacterium* sp.			
		*Leptotrichia* sp.			
		*Actinomyces* sp.	Patchy groups	Observed in the base of the hedgehogs’ structures	
		*Haemophilus/* *Aggregatibacter* sp.	Filamentous	Built a periphery of corncobs structures in addiction with *Streptococcus* sp. cells	
		*Porphyromonas* sp.			
		*Rothia* sp.		Cells of at least four different taxa interact with one another at a micron scale	
		*Lautropia* sp.			
		*Veilonella* sp.			
		*Prevotella* sp.			
		*Neisseria* sp.			

CLSM: confocal laser scanning microscopy; CP: chronic periodontitis; GAP: generalized aggressive periodontitis; NUG: necrotizing ulcerative gingivitis; SUBG-FL: subgingival first layer; SUBG-IL: subgingival intermediate layer; SUBG-OL: subgingival outside layer; SUBG-TL: subgingival top layer; SUBG: subgingival; SUPG-BL: supragingival basal layer; SUPG-SL: supragingival second layer; SUPG: supragingival.

## Data Availability

Not applicable.

## Supplementary Materials

The following are available online at https://www.mdpi.com/article/10.3390/microorganisms9071504/s1: Figure S1: Multiple hedgehogs’ structures adjacent to each other; Figure S2: A cauliflower structure in plaque; Table S1: Database search strategy; Table S2: Oligonucleotide probes’ names and sequences, the microorganisms targeted, and the FISH conditions used in the studies included in this scoping review.
