# Supplementary material for: Friends with Benefits: An Inside Look of Periodontal Microbes’ Interactions Using Fluorescence In Situ Hybridization—Scoping Review
Source: Microorganisms. 2021 Jul 14;9(7):1504. doi: 10.3390/microorganisms9071504 (PMC8306857; doi:10.3390/microorganisms9071504)
Supplement: Supplementary file 1 [file microorganisms-09-01504-s001.zip › microorganisms-1280815-supplementary.pdf]

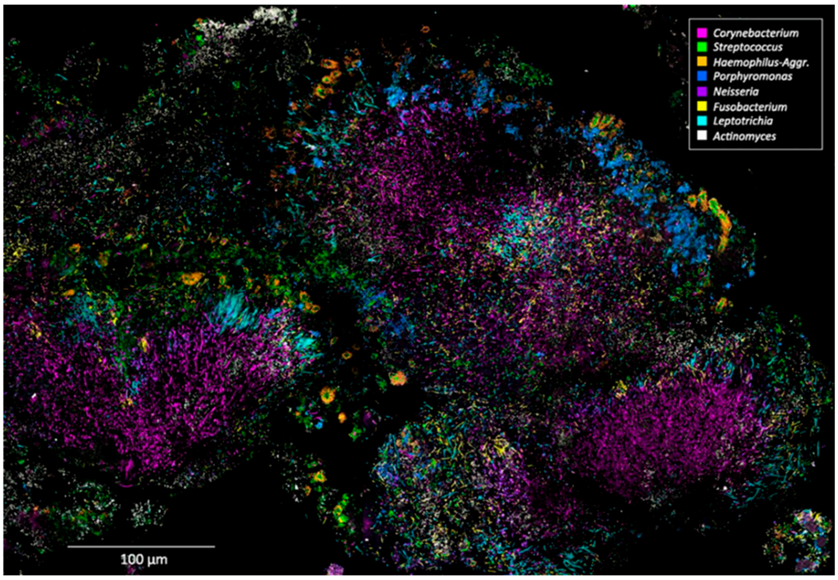


**Figure S1**. Multiple hedgehogs' structures adjacent to each other. Reprinted with the publisher's permission.

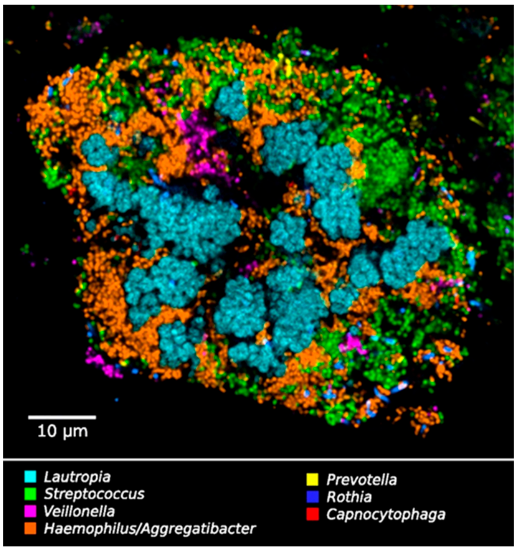


**Figure S2**. A cauliflower structure in plaque. Reprinted with the publisher's permission.

**Table S1.** Database search strategy.

| Database | Search strategy |
|---|---|
| **MEDLINE (via PubMed)** | (Periodont* OR "Periodontal pockets" OR Microbes OR "Oral biofilm" OR Micro* OR "Oral micro*" OR "Bacteria") AND (Imaging OR FISH OR "FISH technique" OR "fluorescence *in situ* hybridization" OR "hybridization") |
| **Scopus (Elsevier**: Amsterdam, The Netherlands**)** | (Periodont* OR "Periodontal pockets" OR Microbes OR "Oral biofilm" OR Micro* OR "Oral micro*" OR "Bacteria") AND (Imaging OR FISH OR "FISH technique" OR "fluorescence *in situ* hybridization" OR "hybridization") |
| **Web of Science (Clarivate Analytics**: Philadelphia, United States**)** | TS = ((Periodont* OR "Periodontal pockets" OR Microbes OR "Oral biofilm" OR Micro* OR "Oral micro*" OR "Bacteria") AND (Imaging OR FISH OR "FISH technique" OR "fluorescence in situ hybridization" OR "hybridization")) |

**Table S2.** Oligonucleotide probes' names and sequences, the microorganisms targeted, and the FISH conditions used in the studies included in this scoping review. The abbreviations can be found at the bottom of the table.

| | | | **Target** | | **FISH conditions** | | |
|---|---|---|---|---|---|---|---|
| **Authors (year)** | **Probe name** | **Sequence (5'→ 3')** | **Taxon** | **Microorganisms** | **% Form. (vol/vol)** | **Hybridization Temp. (°C)** | **Hybidization Time (hours)** |
| **Moter, A., *et al.* (1998)** | EUB338 | GCTGCCTCC CGTAGGAGT | D | *Bacteria* | 0-20 | 46 | 1-3 |
| | TRE I | ACGCAAGCTC ATCCTCAAG | G | Group I of oral treponemes | | | |
| **Wecke, J., *et al.* (2000)** | EUB338 | GCTGCCTC CCGTAGGAGT | D | *Bacteria* | 20 | 46 | 3.5 |
| | TRE I | ACGCAAGC TCATCCTCAAG | G | Group I of oral treponemes | | | |
| **Lepp, P. W., *et al.* (2004)** | SBGA-1 | NAI | S | *Methanobrevibacter oralis* | 0-80 | 65 | 8 |
| **Gmür, R., *et al.* (2004)** | Lbuc668 | TACTCGTGC AGTTCCGTCC | S | *Leptotrichia buccalis Fusobacterium nucleatum* | 30-40 | 46 | 2.5 |
| **Gmür, R. and H. Lüthi-Schaller (2007)** | EUB338 | GCTGCCTC CCGTAGGAGT | D | *Bacteria* | 40 | 46 | 2 |
| | Tfor582 | GCGGACTTA ACAGCCCACCT | S | *Tannerella forsythia* | | | |
| **Drescher, J., *et al.* (2010)** | EUB338 | GCTGCCTC CCGTAGGAGT | D | *Bacteria* | 0-30 | 46 | 3-5 |
| | SELE | TCGGAATGTT GTCCCCATCC | G | *Selenomonas sp. Centipeda sp.* | | | |
| | FUSO | CTAATGGGA CGCAAAGCT CTC | | *Fusobacterium sp.* | | | |
| **Schlafer, S., *et al.* (2010)** | EUB338 | GCTGCCTC CCGTAGGAGT | D | *Bacteria* | 0-30 | 46 | 3-5 |
| | FIAL | TCTTTGTCCAC TATCGTTTTGA | S | *Filifactor alocis* | | | |
| | EUB338 | GCT GCC TCC CGT AGG AGT | D | *Bacteria* | 0-50 | 50 | 3 |

| Authors (year) | Probe name | Sequence (5′→ 3′) | Target | | FISH conditions | | |
|---|---|---|---|---|---|---|---|
| | | | Taxon | Microorganisms | % Form. (vol/vol) | Hybridization Temp. (°C) | Hybidization Time (hours) |
| **Zjinge, V., *et al.* (2010)** | EUK502 | ACC AGA CTT GCC CTC C | | *Eukarya* | 15-45 | | |
| | CAAL | GCC AAG GCT TAT ACT CGC T | S | *Candida albicans* | 30 | | |
| | STR493 | GTT AGC CGT CCC TTT CTG G | G | most *Streptococcus* sp . and some *Lactococcus* sp. | 0 | | |
| | LAB759 | CTA CCC ATR CTT TCG AGC C | | *Lactobacillus* sp., *Ruminococcaceae* sp. and *Pediococcus* sp. | 35 | 46 | |
| | Mmicros1435 | GCC GCC GAT CTA ACC GCA | S | *Parvimonas micra* | 0 | 50 | |
| | Sel1469 | CCA GTC ACC TTC CCC ACC | G | *Selenomonas* sp. but not *S. sputigena* | 30-50 | 46 | |
| | SynA1409 | ACA CCC GGC TCG GGT GGT | | *Synergistetes* group A | 40-50 | | |
| | ACT218 | CGA GCC CAT CCC CCA CCA | | *Actinomyces* sp. | 0 | 50 | |
| | Fus664 | CTT GTA GTT CCG CYT ACC TC | | most *Fusobacterium* sp. | 40 | 46 | |
| | Fnav1254 | CTT CAC AGC TTT GCG ACT C | S | *F. naviforme, F. nucleatum subsp. fusiforme* | 30 | | |
| | Fnuc133c | GTT GTC CCT ANC TGT GAG GC | | *F. nucleatum, F. periodonticum* | | | |
| | SPIRO1400 | CTC GGA TGG TGT GAC GGG CG | F | subgroup of the *Spirochaetaceae* | ND | 50 | |
| | TrepG1 | GAT TCC ACC CCT ACA CTT | S | *T. medium and T. denticola* | 20-50 | 46 | |
| | Td469 | CAT GAC TAC CGT CAT CAA AGA AGC | | *T. denticola* | ND | 50 | |
| | Aa829 | GGG CTA AAC CCC AAT CCC | | *A. actinomycetemcomitans* | | | |
| | Camp655 | CAT CTG CCT CTC CCT YAC | G | *Campylobacter* sp. | 30 | 46 | |
| | CFB935 | CCA CAT GTT CCT CCG CTT GT | | *Cytophaga-Flavobacterium-Bacteroides cluster* | >40 | 50 | |
| | BAC303 | CCA ATG TGG GGG ACC TT | F | *Bacteroidaceae and Prevotellaceae* | 0 | | |
| | Prev394 | GCA CGC TAC TTG GCT GG | G | *Prevotella* sp. | 25 | 46 | |
| | Pi425 | CTT TAC TCC CCA ACA AAA GCA GTT TAC AA | S | *P. intermedia* | 20 | | |
| | Pnig657 | TCC GCC TGC GCT GCG TGT A | | *P. nigrescens* | ≤ 40 | | |

| Authors (year) | Probe name | Sequence (5′→ 3′) | Taxon | Target Microorganisms | FISH conditions % Form. (vol/vol) | Hybridization Temp. (°C) | Hybidization Time (hours) |
|---|---|---|---|---|---|---|---|
| | Tfor582 | GCG GAC TTA ACA GCC CAC CT | | *T. forsythia* | 40 | | |
| | Tfor440 | CGT ATC TCA TTT TAT TCC CCT GTA | | *T. forsythia* | 20 | | |
| | Tfor127 | CTC TGT TGC GGG CAG GTT AC | | *T. forsythia* | 40 | | |
| | Tfor997 | TCA CTC TCC GTC GTC TAC | | *T. forsythia* | 40 | | |
| | Pg477 | CAA TAC TCG TAT CGC CCG TTA TTC | | *P.gingivalis* | 20 | | |
| | Pendo740 | CAG TGT CAG ACG GAG CCT | | *P. endodontalis* | 30-40 | | |
| | Capno371 | TCA GTC TTC CGA CCA TTG | G | *Capnocytophaga* | 0 | 50 | |
| **Machado, F. C., *et al.* (2012)** | Pint649 | GCCGCCRC TGAASTCAAGCC | S | *Prevotella intermedia* | 40 | 42 | left overnight |
| **Mark Welch, J. l., *et al.* (2016)** | EUB338 | GCTGCCTC CCGTAGGAGT | D | *Bacteria* | 20 | 46 | 2-4 |
| | Act381 | CGTCGCTG CATCAGGCTT | P | *Actinobacteria* | 20 | 46 | 2-4 |
| | Act692 | CTGATATCT GCGCATTCC | P | *Actinobacteria* | 20 | 46 | 2-4 |
| | Act476 | ATCCAGCTA CCGTCAACC | G | *Actinomyces* | 20 | 46 | 2-4 |
| | Cor595 | CCGGAATTT CACAGACGACG | G | *Corynebacterium* | 20 | 46 | 2-4 |
| | Cor633 | AGTTATGCC CGTATCGCCTG | G | *Corynebacterium* | 20 | 46 | 2-4 |
| | Rot491 | TAGCCGGC GCTTTCTCTG | G | *Rothia* | 20 | 46 | 2-4 |
| | Cmat175 | ACTAAACCA TGGTCCTATCCG | S | *Corynebacterium matruchotii* | 20 | 46 | 2-4 |
| | Cap371 | TCAGTCTTC CGACCATTG | G | *Capnocytophaga* | 20 | 46 | 2-4 |
| | Prv392 | GCACGCTAC TTGGCTGG | G | *Prevotella* | 20 | 46 | 2-4 |
| | Pg1160 | CCTCACGCC TTACGACGG | G | *Porphyromonas* | 20 | 46 | 2-4 |
| | Str405 | TAGCCGTC CCTTTCTGGT | G | *Streptococcus* | 20 | 46 | 2-4 |
| | Vei488 | CCGTGGCT TTCTATTCCG | G | *Veillonella* | 20 | 46 | 2-4 |
| | Lmir444 | TGGCACAG TCCTTTTCGTTCC | S | *Lautropia mirabilis* | 20 | 46 | 2-4 |
| | Nei1030 | CCTGTGTTA CGGCTCCCG | F | *Neisseriaceae* | 20 | 46 | 2-4 |

| Authors (year) | Probe name | Sequence (5′→ 3′) | Target | | FISH conditions | | |
|---|---|---|---|---|---|---|---|
| | | | Taxon | Microorganisms | % Form. (vol/vol) | Hybridization Temp. (°C) | Hybidization Time (hours) |
| | Pas111 | TCCCAAGC ATTACTCACC | | *Pasteurellaceae* | | | |
| | Fus714 | GGCTTCCC CATCGGCATT | G | *Fusobacterium* | | | |
| | Lep568 | GCCTAGAT GCCCTTTATG | | *Leptotrichia* | | | |

D: Domain; F: Family; G: Genus; NAI: no available information; ND: not determined; P: Phylum; S: Specie.
